# Intermediate endophenotypes and epigenetic mechanisms of psychiatric disorders

**DOI:** 10.1162/IMAG.a.914

**Published:** 2025-10-08

**Authors:** Thomas Leon Kremer, David Antonio Grimaldi, Henri Fleischer, Emanuel Schwarz, Andreas Meyer-Lindenberg, Urs Braun, Heike Tost

**Affiliations:** Department of Psychiatry and Psychotherapy, Central Institute of Mental Health, Medical Faculty Mannheim, Heidelberg University, Mannheim, Germany; German Center for Mental Health (DZPG), partner site Mannheim-Heidelberg-Ulm, Mannheim, Germany; Department of Neuroscience (DNS), Padova Neuroscience Center, University of Padova, Padua, Italy; Hector Institute for Artificial Intelligence in Psychiatry, Central Institute of Mental Health, Medical Faculty Mannheim, Heidelberg University, Mannheim, Germany

**Keywords:** epigenetics, DNA methylation, neuroimaging, psychiatric disorders

## Abstract

Human neuroimaging and epigenetic research stand ready to advance biological psychiatry. Drawing on the concept of *imaging genetics*, the analysis of associations between human neuroimaging and epigenetic data provides an attractive framework for investigating multi-scale biological mechanisms linking the environment to psychiatric risk and protection. A basic assumption is that environmental stress causes epigenetic changes that lead to alterations in cellular ensembles, which in turn can be measured as changes in brain structure and function using human neuroimaging. However, unlike genotypes, epigenetics varies within individuals, between cell types, or over time, and thus caution is required when inferring the immediacy and directionality of observed associations. In this review, we discuss recent advances and challenges to this methodological framework. Future studies should address causal hypotheses and explain within-individual variance in psychopathology through sophisticated contextualization of observed associations and rigorous analyses of longitudinal data. These advances will be critical for developing a comprehensive understanding of the biological contributions to mental health risk and protection.

## 1 Introduction

Understanding the biological mechanisms of mental health risk and resilience has the potential for new therapeutic and preventive strategies in psychiatry (Kandel, 1998). Among the plethora of methodological subfields in biological psychiatry, research focusing on neuroimaging (Finn et al., 2023) and epigenetics (see Box 1) (Konopka & Bhaduri, 2023) has gained particular popularity in recent years.

Box 1. Basics of Epigenetics

The term epigenetics refers to a set of biological processes that regulate gene expression and enable cells to exhibit different phenotypes over time or across tissues despite sharing the same deoxyribonucleic acid (DNA) sequence (Bird, 2007). Key mechanisms of epigenetic regulation include processes that act on transcriptional regulation, on proteins involved in packaging the DNA (e.g., histone modifications), or directly on the DNA sequence (Inbar-Feigenberg et al., 2013). Among the latter, the addition of a methyl group to the 5’ position of a cytosine in the nucleotide sequence, so-called DNA methylation, is a well-studied epigenetic modification that is dynamic over time and responsive to environmental influences such as stress (Moore et al., 2013; Tognini et al., 2015). In humans, approximately 3% of all cytosines are methylated, predominantly at cytosine–phosphate–guanine (CpG) dinucleotides, which are often concentrated in regions known as CpG islands (Nafee et al., 2008). Canonically, genes are silenced by DNA methylation of CpG islands in their promotor region due to reduced accessibility to transcription factors (Suzuki & Bird, 2008). Aberrant DNA methylation has been implicated in the development of many diseases including psychiatric disorders (Robertson, 2005). For this review, we focus on DNA methylation as an exemplary epigenetic modification. However, we acknowledge the importance of other epigenetic mechanisms in the context of mental health risk and protection that are not the scope of this review (Kouter et al., 2023) such as non-coding ribonucleic acid (RNA) or RNA methylation, both of which have been implicated in stress response regulation and depressive disorder (Engel et al., 2018; Issler et al., 2020).

A basic assumption of human neuroimaging in psychiatry is that mental illness is associated with changes in the structure and/or function of neural circuits (Finn et al., 2023) that can be measured using imaging techniques. Human neuroimaging has reached an impressive level of sophistication due to steady methodological improvements over the years (Finn et al., 2023). For example, brain structural alterations in patients with schizophrenia and depression have been meta-analytically confirmed by large consortia studies (Schmaal et al., 2017; van Erp et al., 2018), and the application of machine learning approaches to psychiatric imaging data has gained significant momentum (Quinn et al., 2024). Interdisciplinary approaches that integrate human neuroimaging with (epi)genetic data are well placed to help overcome the risk of misinterpreting the underlying multilevel mechanisms underlying mental health (Finn et al., 2023).

Around 25 years ago, imaging genetics introduced the idea of modeling neural phenotypes as functions of genetic variation to understand a cascade of biogenetic processes leading to behavior via intermediate neuroimaging phenotypes (Egan et al., 2001; Hariri et al., 2002, see discussion in Meyer-Lindenberg & Weinberger (2006)). For example, prefrontal–hippocampal coupling during working memory (Bähner & Meyer-Lindenberg, 2017) has been shown to be altered in schizophrenia (Meyer-Lindenberg et al., 2005) and has been linked to the effects of a genome-wide supported genetic risk variant in *ZNF804A* (Esslinger et al., 2009). Extending the framework of *imaging genetics* to epigenetics is an intuitive next step to gain deeper insights into multiscale biological mechanisms of mental health risk and protection (Nikolova & Hariri, 2015).

The popularity of epigenetic research in psychiatry stems from its compelling framework for explaining the interactive effects of genetic predisposition and environmental influences, such as childhood trauma, on psychiatric risk (Cavalli & Heard, 2019; Klengel & Binder, 2015). For example, genotype-dependent demethylation of *FKBP5* induced by childhood trauma through glucocorticoid exposure (Klengel et al., 2013) contributes to the genotype-dependent effect of childhood trauma on the risk of developing posttraumatic stress disorder (Binder et al., 2008). This offers a compelling example of gene–environment interactions (Klengel & Binder, 2015), as hypothesized in variations of the classic diathesis-stress model of mental health risk and protection (Belsky & Pluess, 2009).

Unlike genetic variants, epigenetic modifications are temporally and spatially dynamic over time and across different cell types (Cavalli & Heard, 2019). Epigenetic data from peripheral samples such as blood or saliva likely differ substantially from the brain (Loyfer et al., 2023). Thus, an observed association can possibly be mediated by a cascade of unknown biological mechanisms with or without involving the central nervous counterpart of the assessed peripheral epigenetic modification. And while genetic variation naturally precedes changes in brain structure and function (Meyer-Lindenberg & Weinberger, 2006), epigenetic modifications can plausibly influence or be influenced by neural phenotypes (Nikolova & Hariri, 2015). Thus, inferring the directionality of observed associations requires additional information beyond cross-sectional correlative analyses. Within-subject epigenetic variation introduces unique methodological and interpretive challenges beyond those encountered in imaging genetics but also opens opportunities to examine within-subject variance in psychopathology and brain phenotypes as a function of epigenetics.

In this review, we provide a brief overview of current efforts to integrate human neuroimaging and peripheral epigenetics in psychiatry. We will discuss hypothesis-driven epigenetic research on specific genes (see Table 1), as well as data-driven approaches that consider the entire epigenome (see Table 2). We will also discuss the necessity to contextualize and infer the directionality of observed associations in order to facilitate a comprehensive understanding of the biological contributions to mental health risk and protection in the future.

**Table 1.** Results from relevant studies on the exemplary candidate genes FKBP5 and SLC6A4.

Reference	Year	Sample size	Sample type	Epigenetic profiling	MRI profiling	Main results
***FKBP5***
(Klengel et al.)	2013	34	Blood	FKBP5 intron 7	HP volume	Lower FKBP5 intron 7 methylation in peripheral blood was significantly correlated with reduced right HP head volume (r = –0.484, p = 0.014).
(Harms et al.)	2017	33	Saliva	FKBP5 promotor and introns 2, 4, 5, 6, 7, and 9	Voxel-wise activity during inhibitory control	FKBP5 intron 5 methylation mediated the association between early life stress and inhibition-related DLPFC activity (r = –0.49, p = 0.005; p_FWE_ < 0.03).
(Tozzi et al.)	2018	106 (56 MDD)	Blood	FKBP5 intron 7	sMRI: Voxel-wise GMV, task-fMRI: IFOG activity during valence recognition	Lower FKBP5 intron 7 methylation across all participants correlated with reduced bilateral IFOG gray matter concentration (Wald χ² = 11.93, p_FDR_ = < 0.01). In MDD patients, lower FKBP5 intron 7 methylation was associated with higher IFGO activation during emotional valence recognition (Wald χ² = 5.58, p = 0.02).
(Chiarella et al.)	2020	45 adolescents (25 MDD)	Saliva	FKBP5 intron 7	sMRI: Voxel-wise GMV, rs-fMRI: ROI-based FC	sMRI: Lower FKBP5 intron 7 methylation was associated with lower gray matter volume in multiple areas (ACC: Wald χ² = 11.00, p_FDR_ = 0.004; frontal operculum: Wald χ² = 12.30, p_FDR_ < 0.001; IFOG: Wald χ² = 9.79, p_FDR_ = 0.007). rs-fMRI: FKBP5 intron 7 methylation interacted with MDD diagnosis on orbitofrontal–rostral prefrontal cortex connectivity (t = 5.04, p_FWE_ = 0.002).
(Muehlhan et al.)	2020	74	Blood	FKBP5 intron 7	MVPA on FC during emotion processing	MVPA identified a cluster in the right rostral ACC/paracingulate gyrus where connectivity patterns varied as a function of *FKBP5* intron 7 methylation (Threshold: p≤0.001, cluster-level FWE-corrected p≤0.05).
(Womersley et al.)	2022	75 adolescents	Saliva	FKBP5 TSS, promotor, distal and proximal TAD, proximal enhancer, introns 2,3,5, and 7	Thalamus volume	Increased FKBP5 intron 7 methylation was negatively associated with right thalamus volume at three CpG sites (35558438: β = –0.35, p_FDR_ = 0.039; 35558566: β = –0.34, p_FDR_ = 0.039; 35558710: β = –0.33, p = 0.057).
(Kremer et al.)	2024	Multiple datasets: 395 (208 for task-fMRI)	Blood	FKBP5 promotor	sMRI: Voxel-wise GMV, task-fMRI: VMPFC seeded FC during emotional face matching	FKBP5 promotor methylation was positively associated with VMPFC volume (t = 3.99, p_FWE_ = 0.039) and with VMPFC–amygdala coupling during emotion processing (t = -3.49, p_FWE_ = 0.015).
**SLC6A4**						
(Dannlowski et al.)	2014	189	Blood	SLC6A4 promotor in *AluJb* element	Voxel-wise GMV	SLC6A4 promoter *AluJb* methylation showed strong association with bilateral HP volume in both samples (right: t = 5.04, p_FWE_ = 0.006; left: t = 4.58, p_FWE_ = 0.044). Bilateral insula (right: t = 5.17, p_FWE_ = 0.003; left: t = 4.62, p_FWE_ = 0.038), right amygdala (as part of a cluster with the right HP), left superior occipital gyrus (t = 4.81, p_FWE_ = 0.017), and right caudate nucleus (t = 4.69, p_FWE_ = 0.029) also positively correlated with *SLC6A4* promoter *AluJb* methylation.
(Nikolova et al.)	2014	Discovery: 80, Validation: 96 adolescents	Discovery: saliva, Validation: blood	SLC6A4 promotor*,* proximal to TSS	Amygdala activity during fearful face processing	Increased SLC6A4 promoter methylation predicted increased threat-related amygdala reactivity (discovery: t = 11.13, p < 0.00001; replication: t = 11.80, p = 1.29*10^-13^).
(Frodl et al.)	2015	60 (25 MDD)	Blood	SLC6A4 promotor	Voxel-wise activation during emotional attention shifting	Higher SLC6A4 promotor methylation positively correlates with left anterior insula activity during processing of negative emotional stimuli (t = 4.54, p_FWE_ = 0.002), negatively correlates with right posterior insula and right inferior frontal operculum activity during attention shifting away from negative stimuli (t = -5.71, p_FWE_ = 0.005) and negatively correlates with pons activity during attention shifting away from positive stimuli (t = -4.51, p_FWE_ = 0.004). SLC6A4 methylation interacted with MDD diagnosis on hippocampal activity elicited by negative emotional stimuli (t = 4.24, p_FWE_ < 0.001).
(Ismaylova et al.)	2017	40	Blood, saliva, buccal	SLC6A4 promotor	sMRI: Voxel-wise GMV, rs-fMRI: RLP seeded FC, task-fMRI: voxel-wise activity during emotion processing	SLC6A4 promoter methylation derived from whole-blood, saliva, and buccal epithelial cells was positively associated with frontal GMV (blood: t = 4.73, p < 0.001; saliva: t = 4.75, p < 0.001; buccal: t = 4.50, p < 0.001). Blood and buccal-derived SLC6A4 promotor methylation showed positive associations with RLP functional connectivity during emotion processing (blood: t = 5.02, p < 0.001; buccal: t = 6.39, p < 0.001), but saliva-derived methylation did not. SLC6A4 promotor methylation was not associated with brain function during emotion processing.
(Swartz et al.)	2017	132 adolescents	Saliva	*SLC6A4* promotor, proximal to TSS	Amygdala activity during fearful face processing	In a longitudinal analysis, greater increases in SLC6A4 promotor methylation were associated with greater increases in threat-related centromedial amygdala reactivity during the same time window (Beta = 0.26, p = 0.04), potentially mediating an indirect effect of lower socioeconomic status on depressive symptoms.
(Chiarella et al.)	2020	45 adolescents (25 MDD)	Saliva	SLC6A4 promotor	sMRI: voxel-wise GMV, rs-fMRI: ROI-based FC	sMRI: SLC6A4 promotor methylation interacts with MDD diagnosis on IFOG volume (Wald χ² = 6.59, p_FDR_ = 0.036). rs-fMRI: SLC6A4 methylation was linked to amygdala–frontal operculum connectivity (t = 4.04, p_FWE_ = 0.041).

We reported only the datasets and results relevant to epigenetic neuroimaging.

ACC = anterior cingulate cortex, CpG = cytosine–guanine dinucleotide, DLPFC = dorso-lateral prefrontal cortex, DNAm = DNA methylation, FC = functional connectivity, GMV = gray matter volume, HC = healthy controls, HP = hippocampus, ICA = independent component analysis, IFGO = inferior frontal orbital gyrus, MDD = major depressive disorder, MRI = magnetic resonance imaging, MVPA = multi-voxel pattern analysis, RLP = right lateral parietal cortex, ROI = region of interest, rs = resting-state, TAD = topologically associating domain, TSS = transcription starting site, VMPFC = ventro-medial prefrontal cortex.

**Table 2.** Results from relevant studies implementing multivariate analyses, including epigenetic clocks.

Reference	Year	Sample size	Epigenetic profiling	MRI profiling	Main results
**Multivariate analyses**					
(Freytag et al.)	2017	Discovery: 533 young adults, validation: 596	ICA on whole-genome methylation data	Whole-brain CT	One of the components was positively correlated with chronological age (p_FWE_ = 2.31e^—11^) and negatively with CT (p_FWE_ = 5.79e^—7^), both replicable in the validation sample (p < 10e^—10^). The epigenetic signature was also found to partially mediate the effect of age on CT (p < 0.001), and to negatively correlate with episodic memory.
(Lohoff et al.)	2021	Discovery: 539 (336 AUD) Replication 1: 86 (43 AUD) Other: 4798	EWAS of AUD	sMRI: HP volume task-fMRI: fear conditioning	After FDR correction, 96 probes were significant in the first two cohorts. 70 were associated with AUD in ≥ 1 additional cohort. AUD-associated DNA methylation patterns were linked to increased right hippocampal volume (p=0.007) and reduced activity in bilateral amygdala and insula during fear conditioning (p≤ 0.05).
(Chen et al.)	2020	Multiple datasets: 2041 (803 SCZ) Postmortem: 244 (108 SCZ)	MRS for SCZ on blood data, validated on the postmortem sample	DLPFC-HP FC during WM task	The MRS was associated with SCZ across the three datasets and in postmortem DLPFC. In HCs, it was negatively correlated with DLPFC-HP FC (intermediate phenotype of SCZ) across two MRI datasets (voxel–level p_FWE_ < 0.04 corrected for bilateral HP, and p_FWE_ = 0.02 corrected for the subregion where the first region was located).
(Chen et al.)	2021	Multiple datasets: 1198 Postmortem: 244 samples (108 SCZ)	CCA performed on the CpGs of each pair of genes	DLPFC-HP FC during WM task	The pairwise correlations were organized in a connected network in samples 1 and 2. The network structure was replicated in the DLPFC, where SCZ showed reduced mean coordination. In sample 3, the mean coordination predicted increased DLPFC-HP FC (voxel-level p_FWE_ < 0.05 across bilateral HP).
(Jia et al.)	2021	3337	Meta-analysis of EWAS and DMR analyses of MRI phenotypes	Volume of thalamus, HP, and nAcc	EWAS revealed two loci associated with HP volume at p_FDR_ < 0.05 (cg26927218 and cg17858098). Evidence of DMRs linked to HP volume (Šidák p < 0.05), affecting the expression of genes involved in learning and memory.
(Hu et al.)	2022	38 FES before and after 8 weeks of treatment with risperidone, 38 HC	EWAS pre- vs post-treatment, comparison vs HC at both time points	rs-fMRI measures altered by treatment (ALFF/ReHo of striatum)	Nominally significant changes were observed in ~6000 methylation sites. Among sites with beneficial effect, 580 were normalized by treatment. In 122 of them, the changes were correlated with fMRI phenotypes (p < 0.05).
(Sun et al.)	2023	Discovery: 506 adolescents Validation: 823 adolescents	Cross-sectional and longitudinal mixed linear EWASs, MRSs of EB and IB in adolescents	Voxel-wise GMV	Cross-sectional EWASs identified no results, while longitudinal EWAS of EB identified 1 locus (cg01460382, IQSEC1). MRSs for EB effectively predicted EB in cross-validation models and were linked with reduced GMV in regions including ACC/MCC, middle OFC, and fusiform gyrus (p_FWE_ < 0.05), which partly mediated the association between MRS and EB (p < 0.05).
(Yang et al.)	2023	9732 middle-aged to older individuals	EWAS and DMR analysis of MRI phenotype	WMHs burden	WMHs, a marker of cerebral small vessel disease, were associated with 12 single CpG (p_FWE_ < 0.05, top locus was cg24202936, associated with coagulation factor II expression in the blood), and with DMRs in PRMT1 and CCDC144NL-AS1 (p < 0.05 corrected using multiple methods).
**Epigenetic clocks**					
(Raina et al.)	2017	713	Hannum and Horvath clocks	WMH burden	DNAm acceleration was associated with WMH burden even after accounting for the crucial risk factors for atherosclerosis, including BMI, BP, diabetes, and smoking status (p < 0.05).
(Proskovec et al.)	2020	79	Consensus clock (Hannum, Horvath)	CT (vertex-wise analysis)	DNAm acceleration was associated with reduced CT including OFC and temporal areas (p < 0.01, cluster restriction based on GRF theory).
(Wiesman et al.)	2020	68	Consensus clock (Hannum, Horvath)	Flanker task during MEG, co-registered with MRI	DNAm age predicted selective attention and fully mediated its link with chronological age. DNAm acceleration predicted increased gamma activity in the ACC during selective attention, reflecting heightened neural resources recruitment (voxel-level p_FWE_ < 0.05).
(Cheong et al.)	2022	79	Horvath clock	CT, SA, and volume of 148 brain regions	DNAm acceleration was associated with reduced volume of the right cuneus gyrus (q_FDR_ = 0.04).
(Milicic et al.)	2022	Discovery: 373 Validation: 486	Hannum, Horvath, PhenoAge, Zhang EN, Zhang BLUP clocks	HP cortical volume	Associations between DNAm acceleration and cognition or Aβ were not detected, while DNAm acceleration according to Hannum clock was associated with HP cortical volume in the discovery cohort (p_FDR_ < 0.05) and subgroups of the validation cohort (p < 0.05).
(Nishitani et al.)	2022	51 mothers, 30 with MRI	Horvath clock	Voxel-wise GMV	Decreased DNAm acceleration was linked to reproductive effort and to increased GMV in the left precuneus (p_FWE_ = 0.04), which mediated the relationship between parity status and DNAm acceleration.
(Whitman et al.)	2024	Cohort 1: 770 Cohort 2: 903 Cohort 3: 649	DunedinPACE	Brain volume, HP volume, WMH volume, CT, SA	Across all the three datasets, DNAm acceleration was related to lower brain volume, HP volume and CT, and higher WMH volume (p < 0.05). In one dataset, it also predicted reduced SA (p=0.01).
(Graves et al.)	2024	103	GrimAge clock	CPM on rs-fMRI FC data	Individuals with accelerated epigenetic age performed worse across multiple cognitive domains. CPM predicted DNAm acceleration, revealing a pivotal role of the medial temporal and limbic regions.
(Newman et al.)	2025	98, of which 41 at 2 years follow-up	GrimAge clock	Brain volume, WMH volume, and microstrucure	DNAm acceleration significantly predicts brain volume, WMH volume and microstructural composition (p < 0.01), as well as the microstructural composition longitudinal changes (p < 0.05).
(Sant’Anna Barbosa Ferreira et al.)	2025	254 (twin pairs)	Hannum, Horvath, PhenoAge, GrimAge, DunedinPACE clocks	Brain age prediction based on structural MRI	DNAm acceleration according to Hannum (p_FWE_ = 0.033) and GrimAge (p_FWE_ = 0.015) predicts brain age acceleration. The association was poorly explained by genetic and shared environmental factors, suggesting a strong role of unique environmental factors.

We reported only the datasets and results relevant to epigenetic neuroimaging. All epigenetic data are obtained from blood samples.

Aβ = amyloid beta, ACC = anterior cingulate cortex, ALFF = amplitude of low-frequency fluctuations, AUD = alcohol use disorder, CCA = canonical correlation analysis, CPM = connectome-based predictive modeling, CT = cortical thickness, DLPFC = dorso-lateral prefrontal cortex, DMRs = differentially methylated regions, DNAm = DNA methylation, EB = externalizing behaviors, EWAS = epigenome-wide association study, FC = functional connectivity, GMV = gray matter volume, HC = healthy controls, HP = hippocampus, IB = internalizing behaviors, ICA = independent component analysis, MCC = middle cingulate cortex, MRI = magnetic resonance imaging, MRS = methylation risk score, nAcc = nucleus Accumbens, OFC = orbitofrontal cortices, ReHo = regional homogeneity, rs-fMRI = resting-state functional magnetic resonance imaging, SA = surface area, SCZ = schizophrenia, WMH = white matter hyperintensity.

## 2 Current State of the Field

**Fig. 1.**
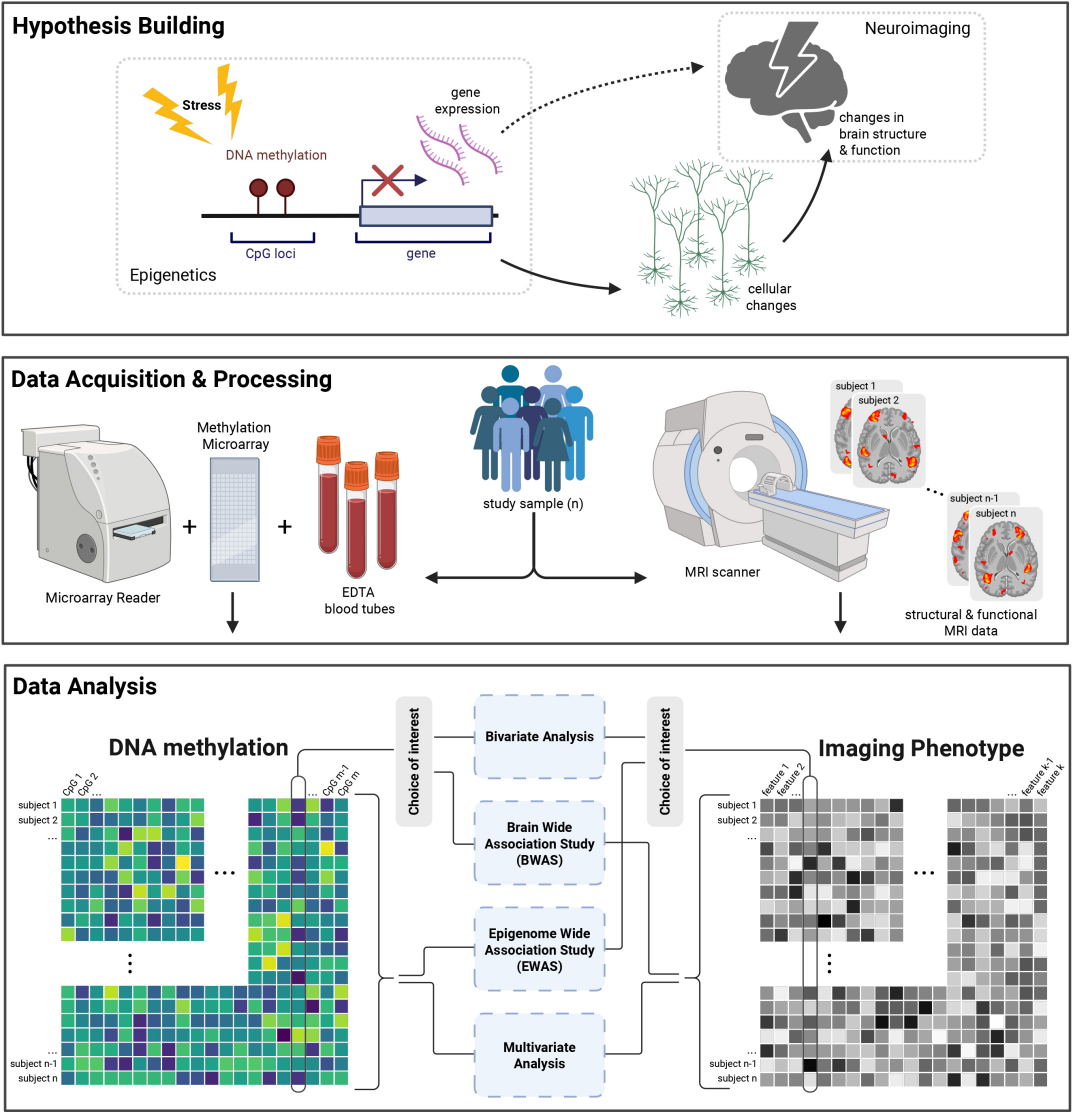
Conceptual illustration of combining human neuroimaging with peripheral epigenetic data to address hypotheses about biological contributions to psychiatric disorders. Created in BioRender. Kremer, T. (2025) https://BioRender.com/5iajrgn

In 2011 an Italian research group was the first to examine neural correlates of peripheral epigenetics in humans by showing that stress-related DNA methylation of the *COMT* Val158 allele in blood correlates with prefrontal cortical efficiency and working memory performance (Ursini et al., 2011). Here, a link is drawn from stressful life events over specific epigenetic modifications and a neural phenotype to impaired cognitive performance. A basic assumption is that environmental stress causes epigenetic changes that lead to alterations in cellular ensembles, which in turn can be measured as changes in brain structure and function using human neuroimaging (as depicted in Fig. 1A). To study this assumption, epigenetic data can be acquired using DNA methylation microarray technology on whole blood samples and brain scans using structural or functional magnetic resonance imaging (as depicted in Fig. 1B). Hypothesis-driven feature selection allows to test for a statistical association between an epigenetic and a neuroimaging phenotype of interest. However other methods of dimensionality reduction are feasible (see Fig. 1C). Based on previous findings from *imaging genetics*, Nikolova et al. (2014) showed that higher methylation of *SLC6A4*, encoding a serotonin transporter, relates to increased amygdala activity during emotion processing (Nikolova et al., 2014). Changes in *SLC6A4* methylation mediate the effect of socioeconomic status on amygdala activity (Swartz et al., 2017).

### 2.1 The FKBP5 model

*FKBP5* is involved in molecular stress signaling and offers a prime example of the method of linking peripheral epigenetics and neuroimaging phenotypes (Matosin et al., 2018). The epigenetic signature of FKBP5 mediates gene–environment interactions on psychiatric risk after early life stress via glucocorticoid-dependent DNA demethylation (Klengel et al., 2013; Lee et al., 2011; Zannas et al., 2019). In several studies, demethylation of *FKBP5* in peripheral blood was associated with reduced gray matter volume in the hippocampus or frontal cortex (Klengel et al., 2013; Kremer et al., 2024; Tozzi et al., 2018), potentially because *FKBP5* methylation moderates the neurostructural effects of glucocorticoid exposure on the brain areas involved in the neuroendocrine axis (Kremer et al., 2024). In functional analyses, *FKBP5* demethylation mediates the effect of early life stress on inhibition-related prefrontal activity (Harms et al., 2017) in emotion regulatory brain circuits (Kremer et al., 2024; Muehlhan et al., 2020) implicated in the development of stress-related psychiatric disorders (Herringa et al., 2016; Kremer et al., 2024; Liu et al., 2020; Meyer-Lindenberg & Tost, 2012). The example of *FKBP5* shows how translational findings from basic research in humans and animals can reveal a paradigmatic signaling pathway that explains, at least in part, the development of a psychiatric risk constellation from early childhood stress to epigenetic modifications and neuronal changes (Matosin et al., 2018).

The candidate gene approach requires an *a priori* hypothesis from preexisting knowledge, is statistically efficient, and can yield interpretable results on genes that may eventually qualify as drug targets (Tabor et al., 2002). However, it has received legitimate criticism regarding its low replicability (Border et al., 2019), primarily because studies with small sample sizes are not adequately powered for the small effects of common genetic risk variants on complex phenotypes (Marek et al., 2022). Thus, adequate power, transparent methods, and independent replications are necessary for enhanced replicability (Carter et al., 2017).

### 2.2 Epigenome-wide analyses

Epigenome-wide association studies (EWASs) do not require a hypothesis-based selection of candidate genes, but rather large samples and sufficient computational power. The ENIGMA consortium performed an epigenome-wide meta-analysis of association studies on hippocampus, thalamus, and nucleus accumbens volumes in a total sample of 3337 individuals (Jia et al., 2021). Only hippocampal volume had a significant association with DNA methylation at two CpG sites at the genome-wide significance level (Jia et al., 2021). The identified methylation sites are thought to regulate the expression of a synaptic protein involved in learning and memory functions, as well as an enzyme involved in fat metabolism. Although not self-explanatory, the identified epigenetic association can inform hypothesis building for follow-up studies, such as those investigating the mechanistic influence of lifestyle factors on hippocampal structure and cognition. A longitudinal EWAS of adolescents at 14 and 19 years of age identified methylation patterns associated with externalizing behavior and reduced gray matter volume in orbitofrontal and anterior cingulate cortical areas (Sun et al., 2023). Interestingly, using longitudinal data, the authors showed that these methylomic changes were the result of externalizing behavior, intensified by negative life events, rather than the cause of it (Sun et al., 2023). An EWAS of white matter lesions, a neuroimaging phenotype linked to dementia and stroke, revealed significant associations with a dozen single CpG sites and more differentially methylated regions (Yang et al., 2023). Mendelian randomization suggested a potential causal effect of DNA methylation at these sites on developing white matter lesions (Yang et al., 2023). An analysis by Freytag et al. (2017) used independent component analysis on methylome data to identify components that mediate the effect of age on cortical thickness. This work highlights the potential of reducing the dimensionality of methylation data by associating epigenetics with a variable of interest, such as age (see Box 2) (Graves et al., 2024). In 2020, Chen et al. (2020) trained a classifier on blood methylation data to predict schizophrenia diagnosis in a large sample of 2041 individuals across 3 cohorts, which showed promising areas under the curve ranging from 69 to 78.2. They identified immune-associated, neural, and synaptic pathways as key contributors (Chen et al., 2020). A risk score based on this classifier correlated with altered prefrontal–hippocampal coupling during working memory (Chen et al., 2020), the beforementioned established intermediate phenotype for schizophrenia (Bähner & Meyer-Lindenberg, 2017). The same research group associated the same neuroimaging phenotype with hyper-coordinated methylation patterns involved in synaptic processes (Chen et al., 2021). These results demonstrate the intricate links between peripheral epigenome-wide methylation characteristics and neuroimaging in the context of psychiatric phenotypes.

Box 2. Epigenetic Aging

Age strongly affects DNA methylation (Horvath, 2013) and can by estimated by applying machine learning approaches to methylation data (Horvath & Raj, 2018; Rutledge et al., 2022). Some measures of mismatch between *epigenetic clocks* and chronological age, acceleration, or deceleration, have been shown to predict mortality risk and a range of age-related diseases (Horvath & Raj, 2018). Discrepancies between epigenetic and chronological age have also been linked to numerous psychiatric disorders (Higgins-Chen et al., 2020; Protsenko et al., 2021) and to cognitive function (Graves et al., 2024; Hillary et al., 2021). Structural neuroimaging studies have demonstrated associations between accelerated epigenetic aging and increased white matter hyperintensities, reduced whole brain and hippocampal volume (Hillary et al., 2021; Milicic et al., 2022; Whitman et al., 2024), and regional reductions in cortical thickness and surface area (Cheong et al., 2022; Proskovec et al., 2020). In 2017, Freytag et al. identified a specific methylation signature that mediates the effect of age on cortical thinning and correlates with lower memory performance (Freytag et al., 2017). Using connectome-based predictive modeling, Graves et al. (2024) recently showed that functional connectivity of the medial temporal lobe and limbic regions mediates the relationship between epigenetic age acceleration and impaired cognitive performance in seniors, paving the way for deeper research into the relationship between brain function and epigenetic aging. Recently, a Dutch twin study showed relationships between brain age acceleration and epigenetic age acceleration based on the Hannum and GrimAge clock (Sant’Anna Barbosa Ferreira et al., 2025), after some other studies did not observe such an association using other epigenetic clocks such as the Horvath clock (Mareckova et al., 2023; Teeuw et al., 2021). This implicates that some epigenetic clocks capture biological processes distinct from brain structural aging, while Hannum and GrimAge, however, seem to capture a more systemic aging process present in brain structure and peripheral DNA methylation that might have an environmental etiology (Sant’Anna Barbosa Ferreira et al., 2025) but requires further investigation.

## 3 Perspective for Future Research

Future research should target mental health risk and protection with a particular emphasis on specific biological hypotheses and the elucidation of the points of convergence and divergence of neural and epigenetic risk mechanisms. Here, naturally, biological psychiatry should prioritize research with translational potential to enhance prevention and therapeutic outcomes (Kandel, 1998). Specifically, elucidating putative neuroepigenetic pathways through which environmental exposures, such as early-life stress or urbanicity, confer risk for psychosis may yield important mechanistic insights and novel targets for prevention (Cheng et al., 2021; Lederbogen et al., 2011). Moreover, the systematic investigation of the effects of antipsychotic medication on neuroepigenetic processes and their therapeutic relevance will be essential to advance efforts in treatment stratification and optimization (Davyson et al., 2025).

Concerning technical needs, future research integrating human neuroimaging with peripheral epigenetics will require sufficiently large sample sizes and out-of-sample replications to find robust associations (Campagna et al., 2021; Marek et al., 2022). ENIGMA (Jia et al., 2021) and MIND (Schuurmans et al., 2024) are two international and large consortia sampling both modalities that will create valuable datasets.

Handling those high-dimensional datasets requires analytical methods equipped for this computationally demanding task, such as machine learning approaches (Bzdok & Meyer-Lindenberg, 2018). In brain cancer diagnostics, the application of machine learning on DNA methylation of tumor biopsies has already been successfully translated into clinical practice (Capper et al., 2018). *Epigenetic clocks* (see Box 2) are another successful example of applying machine learning on methylation data to estimate biological aging (Horvath, 2013). The strategy to predict some relevant variable, such as aging or diagnosis, from methylation is dependent on the traceability of this variable in the methylome. In 2020, Chen et al. have applied this strategy to classify schizophrenia patients from methylation data and associated the resulting polygenic methylation risk score with an intermediate neuroimaging phenotype implicated in psychosis (Chen et al., 2020). Psychopharmacology exposure leaves a trace on the methylome and might qualify as a relevant variable to predict from methylation data in future studies (Davyson et al., 2025). Machine learning approaches equipped to find associations between two high-dimensional vectors such as partial least squares or canonical correlation analysis would preserve the full data structure on both ends of this multimodal integration, but they have not yet been applied to peripheral epigenetics and human neuroimaging (Mihalik et al., 2022). It also remains to be studied whether machine learning models trained on neuroimaging and epigenetic data could improve the prediction of clinically relevant variables such as disease trajectory or treatment response.

**Fig. 2.**
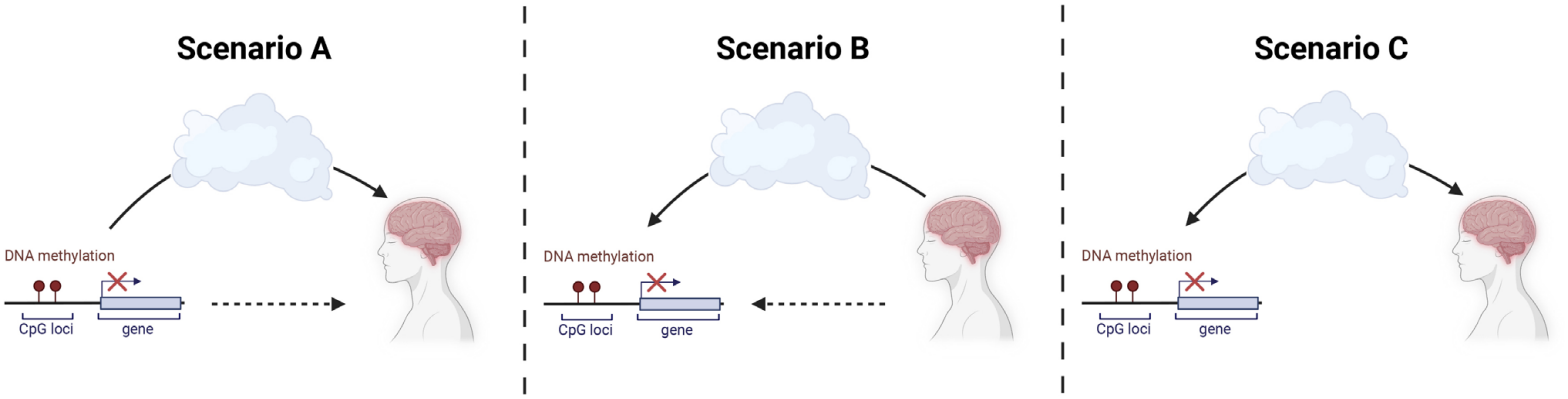
Overview of the various, possibly overlapping, scenarios underlying a statistical association between peripheral epigenetics and neuroimaging phenotypes. Created in BioRender. Kremer, T. (2025) https://BioRender.com/xib12dm

More specific areas of tension for this method arise from the variance of methylation over time or between cell types (Cavalli & Heard, 2019; Zaimi et al., 2018), which makes it difficult to interpret directionality and immediacy (Nikolova & Hariri, 2015). In principle, three scenarios can explain an observed association between peripheral epigenetic variance in the blood and a neuroimaging phenotype (Fig. 2). In scenario A, changes in peripheral DNA methylation might cause a neuroimaging phenotype, through direct or indirect mechanisms (Fig. 2). This scenario reflects the intuitive hypothesis that epigenetic modifications lead to cellular changes that are then measured as variation in neuroimaging phenotypes. Scenario B, in contrast, is that a neural phenotype might induce changes in DNA methylation, again through direct or indirect mechanisms (Fig. 2). For example, differences in hypothalamus functioning can plausibly lead to changes in DNA methylation by influencing glucocorticoid excretion (Provençal et al., 2020). In scenario C, there is no causal relationship between the intercorrelated epigenetic and neuroimaging phenotypes, but their association is confounded by a third direct or indirect variable (Fig. 2). Aging-associated processes broadly influence DNA methylation (Horvath, 2013) and many imaging phenotypes (Bethlehem et al., 2022) can confound an association, if the analysis does not sufficiently account for this possibility. In patient populations, the effect of psychopharmacological medication on both DNA methylation (Swathy & Banerjee, 2017) and imaging phenotypes (Tost et al., 2010) can be a confounder that needs to be accounted for. Due to the ethical limitations of human experiments, it is nearly impossible to directly test these scenarios, but we will discuss two explicit recommendations to maximize evidence for one or a mixture of these scenarios.

### 3.1 Contextualization of phenotypes

Knowing more about the phenotypes involved in an observed transmodal association helps to infer the underlying mechanism. This can be done by contextualizing the epigenetic modification on the one hand and the neuroimaging phenotype on the other. Downstream effects of epigenetic modifications on gene expression can be assessed by additional wet-laboratory experiments or by exploiting publicly available databases on the functional properties of the human epigenome, for example, from the Human Epigenome Project (https://egg2.wustl.edu/roadmap/web_portal/). An economical way to gain valuable information at low cost is to use publicly available datasets. For example, blood–brain concordance of specific CpG sites may be informative about the immediacy of an observed association and can be assessed using several web applications based on *postmortem* or *ex vivo* (neurosurgical) brain samples (Braun et al., 2019; Edgar et al., 2017; Nishitani et al., 2023). However, low blood–brain concordance does not necessarily preclude a meaningful association between peripheral epigenetics and neuroimaging phenotypes that can plausibly arise due to indirect mechanisms (Nikolova & Hariri, 2015). Conversely, epigenetic loci that are involved in neurodevelopment, tissue differentiation, and neural functioning are more likely to differ in their methylation between blood and brain (Davies et al., 2012) and are of particular interest in psychiatric research.

Similarly, involved neuroimaging phenotypes can be characterized by using neuropsychological variables acquired in the study sample and demonstrating behavioral, cognitive, or affective involvement and relevance. By harvesting public databases such as *Neurosynth* (https://neurosynth.org/) researches can access a sheer mass of exciting brain maps from delicate analyses on brain structure, function, metabolism, chemistry, and so on (Markello et al., 2022). Analogous to the concept of brain mapping, a statistical map of association statistics between a neuroimaging phenotype and peripheral epigenetics can be tested for specific features underlying an observed association. For contextualizing neural correlates of peripheral epigenetic variation, the publicly available Allen Human Brain Atlas (Hawrylycz et al., 2012) offers brain-wide coverage of gene transcription from six postmortem brains. For example, when examining the brain structural correlates of *FKBP5* methylation, the identified ventromedial prefrontal cortex region collocated with the region with the lowest FKBP5 expression levels (Kremer et al., 2024). This contextualization enables careful speculation on a central nervous mechanism through which lower *FKBP5* expression in a brain region enables closer neuroendocrine coupling and thus stronger glucocorticoid-dependent neural effects in this brain area. Through extensive contextualizing of an observed transmodal association, a more educated interpretation of directionality and immediacy is possible.

### 3.2 Chances of within-individual epigenetic variation

While genetics varies between individuals, epigenetics varies between individuals and within individuals (Cavalli & Heard, 2019). Temporal variability of epigenetics hampers interpreting directionality of observed associations and with temporality being a key Bradford Hill criterion, it hampers causal inference (Hill, 1965). Also, due to temporal dynamics, epigenetics can have within-individual effects on changes in psychopathology, for example, the onset-risk or resolution-likelihood of a psychotic episode (Kebir et al., 2017).

Hypothetically, environmental stress induces epigenetic modifications that lead to changes in cellular ensembles, measured as alterations in brain structure and function using human neuroimaging (Fig. 1A). But the case of reverse directionality, that is, psychopathology or neuroimaging phenotypes leading to DNA methylation changes, must not be overlooked, as, for example, in the metabolic consequences of dietary changes (Niculescu & Zeisel, 2002), alcohol use (Liu et al., 2018), or smoking (Zeilinger et al., 2013) in the presence or absence of psychopathology (Farris et al., 2017).

In cross-sectional datasets, instrumental variables can help to infer temporality by triangulation (Marinescu et al., 2018). The concept of Mendelian randomization is based on strong genetic influences on many neuroimaging phenotypes and DNA methylation to use genetics as instrumental variable (Guo et al., 2022; Relton & Davey Smith, 2015; Richmond et al., 2016). Yang et al. (2023) used Mendelian randomization to suggest a causal effect of DNA methylation at specific loci on MRI-based white matter hyperintensities.

Longitudinal sampling schemes with epigenetics and neuroimaging at multiple time points can improve replicability by increasing standardized effect sizes and allow to disaggregate between-individual and within-individual effects (Kang et al., 2024; Montoya, 2023). These can differ substantially and need to be modeled explicitly in a multilevel model (Curran & Bauer, 2011). In theory, the same epigenetic modification might have contrary neural effects between-individual due to neurodevelopmental effects before sampling and within-individual due to neuroplastic effects between time points (Keverne et al., 2015; Tognini et al., 2015). With longitudinal data, the temporal sequence of an observed association can be directly assessed by estimating the predictive power of each associated variable for future values of the other (Richmond et al., 2016; Shojaie & Fox, 2022). Step-by-step the analyzed neuroepigenetic mechanisms can be formulated into a grander biological cascade mediating genetic and environmental influences on mental health risk and protection (Hayes, 2017; Meyer-Lindenberg & Weinberger, 2006).

Causally understanding the contributing biological cascades can enable treatment stratification, preventive strategies, and the identification of novel drug targets to benefit psychiatric patients (Michoel & Zhang, 2023).

## 4 Summary and Conclusion

In this review, we discussed recent advances in combining human neuroimaging with peripheral epigenetic data to study multiscale biological mechanisms of mental health risk and protection. We have highlighted examples of hypothesis-driven and data-driven approaches to exploit these highly compelling and often high-dimensional data modalities. Evidence suggests that epigenetic changes at key loci involved in molecular stress signaling mediate the effect of early life stress on imaging phenotypes involved in affective processing relevant to psychiatric risk (Klengel et al., 2013; Kremer et al., 2024). We then provided a perspective on current areas of tension to explore specific pitfalls and opportunities for future scientific endeavors in this area. In future, it will be important to dissect the precise directionality and pathways of epigenetic mechanisms on neural phenotypes in the context of mental health risk and protection. Understanding bidirectional links between epigenetics and neural structure or function in mental health risk and protection could help to discover novel therapeutic or even preventive strategies to benefit patients.

## Data Availability

No data was acquired or analyzed for this review.
